# Complementary Employment of Shell DFT-1/2 and HSE06 for Defect State Calculations in InP

**DOI:** 10.3390/ma19173577

**Published:** 2026-08-23

**Authors:** Zeliang Liu, Jiangzhen Shi, Hongjing Lai, Shanzhong Xie, Qin Xu, Kan-Hao Xue

**Affiliations:** 1 Jiujiang University, Jiujiang 332005, China; aptblaze@163.com (Z.L.);; 2 School of Integrated Circuits, Huazhong University of Science and Technology, Wuhan 430074, China

**Keywords:** shell DFT-1/2, hybrid functional, point defects, InP, electronic structures

## Abstract

Defect calculations for semiconductors demand both large supercells and accurate electronic structures, posing a significant challenge to first-principles methods. Conventional density functional theory (DFT) with local or semi-local exchange-correlation functionals severely underestimates the band gap, whereas hybrid functionals such as HSE06 provide higher accuracy but at a substantially increased computational cost. In this work, we demonstrate that shell DFT-1/2, a self-energy correction method for electronic structure calculations, may be used jointly with HSE06 to reach the optimal efficiency as well as accuracy. In particular, indium phosphide (InP) was taken as an example. The shell DFT-1/2 method was utilized to yield accurate band structures with a 1.44 eV direct gap, without any empirical parameter. Subsequently, the portion of exact exchange was tuned to match the shell DFT-1/2 electronic structure in HSE06 calculations. The charge transition levels of various point defects in InP were derived using HSE06, and HSE06 and shell DFT-1/2 may be employed alternatively to yield the density of states for the defective supercells. Their consistency proves the feasibility of the complementary employment of the two methods, and this strategy is readily extendable to other semiconductor research.

## 1. Introduction

Indium phosphide (InP), as a typical III–V compound semiconductor, possesses a direct band gap, high electron mobility, excellent photoelectric conversion efficiency, good thermal conductivity, and strong radiation resistance. It is a core functional material for high-speed optical communications, photodetectors, LiDAR, solar cells, and fiber-optic sensors, and plays an irreplaceable role in frontier fields such as 5G communications, artificial intelligence, biomedical imaging and environmental monitoring [1,2,3]. Electronic structure constitutes the primary physical foundation for understanding the optoelectronic properties of semiconductors. Early experiments determined the bulk band gap of InP through optical absorption, photoluminescence, and reflectance spectroscopy [4,5,6]. Yet, resolving detailed band-structure features throughout the Brillouin zone seems difficult by experiments alone. With the development of computational materials science, first-principles calculations exemplified by density functional theory (DFT) have become a central tool in modern computational materials research, enabling atomistic prediction of electronic structures, geometrical configurations, and physicochemical properties [7,8,9].

A central topic for semiconductors such as InP concerns intrinsic point defects, including vacancies, interstitials, and anti-sites, which are unavoidable microscopic defects formed during material growth and operation. They directly determine carrier concentration, mobility, leakage current, and device reliability [10]. For InP, several theoretical and experimental studies have investigated defect behaviors and the associated electrical effects. Vedel et al. performed numerical simulations using Synopsys QuantumATK to explore how defect introduction modulates the resistance and current–voltage characteristics of InP [11]. Sun et al. investigated the impact of defects on InP-based high-electron-mobility transistors through numerical simulation [12]. Experimentally, Chen et al. successfully introduced P_In_ anti-site defects during the low-temperature growth of InP at 60–350 °C by gas-source molecular beam epitaxy, thereby realizing n-type doping [13]. Theoretically, Seitsonen employed the first-principles Car–Parrinello method to optimize the valence electronic structures and ionic positions of vacancies and anti-sites in InP, revealing that In vacancies and P anti-sites dominate under P-rich conditions (P vacancies also have a low formation energy), whereas P vacancies are predominant under In-rich conditions [14]. However, early theoretical calculations were limited by the accuracy of the employed functionals. Schmidt et al. calculated the electronic and structural properties of various P_In_ anti-site defects, but obtained a band gap of only 1.05 eV, which deviates from the experimental value of 1.42 eV [15]. Although Liu et al. managed to enlarge the calculated band gap to 1.32 eV using a finite-size-corrected hybrid functional approach, the high computational cost renders it still difficult to carry out large-scale defect supercell studies [16].

While existing studies have provided basic understanding of defects in InP, an important limitation remains. On the one hand, conventional DFT functionals, within either local density approximation (LDA) or generalized gradient approximation (GGA), severely underestimate the band gap. On the other hand, hybrid functionals, despite their higher accuracy, are computationally expensive. Hence, an electronic-structure calculation scheme that balances accuracy and efficiency is still lacking. In this work, we alternatively employ the shell DFT-1/2 method and the Heyd–Scuseria–Ernzerhof (HSE06) functional [17,18] in InP defect research, which reaches a delicate balance between computational efficiency and accuracy. In particular, the shell DFT-1/2 calculation shows a time cost comparable to that of conventional GGA. And the paper is organized as follows. First, we discuss the similarity of these two methods, and explain why they could be used interchangeably for certain purposes. Then, the two methods are applied to investigate the defect levels in InP. Subsequently, limitations of the shell DFT-1/2 approach in semiconductor defect studies will be explored. With an adaptive interchange between shell DFT-1/2 and hybrid functionals, the computational efficiency for defects in semiconductors can be greatly optimized, while maintaining the empirical parameter-free feature during calculation. While this work does not involve a new functional or a new theory, it emphasizes the significance of self-interaction elimination in electronic structure computation, and shows the feasibility of shell DFT-1/2 to partly replace hybrid functional calculations for electronic structures.

## 2. Theoretical Basis and Rationale for Interchangeability Between Shell DFT-1/2 and HSE06

### 2.1. The Hybrid Functional Approach

**Figure 1:**
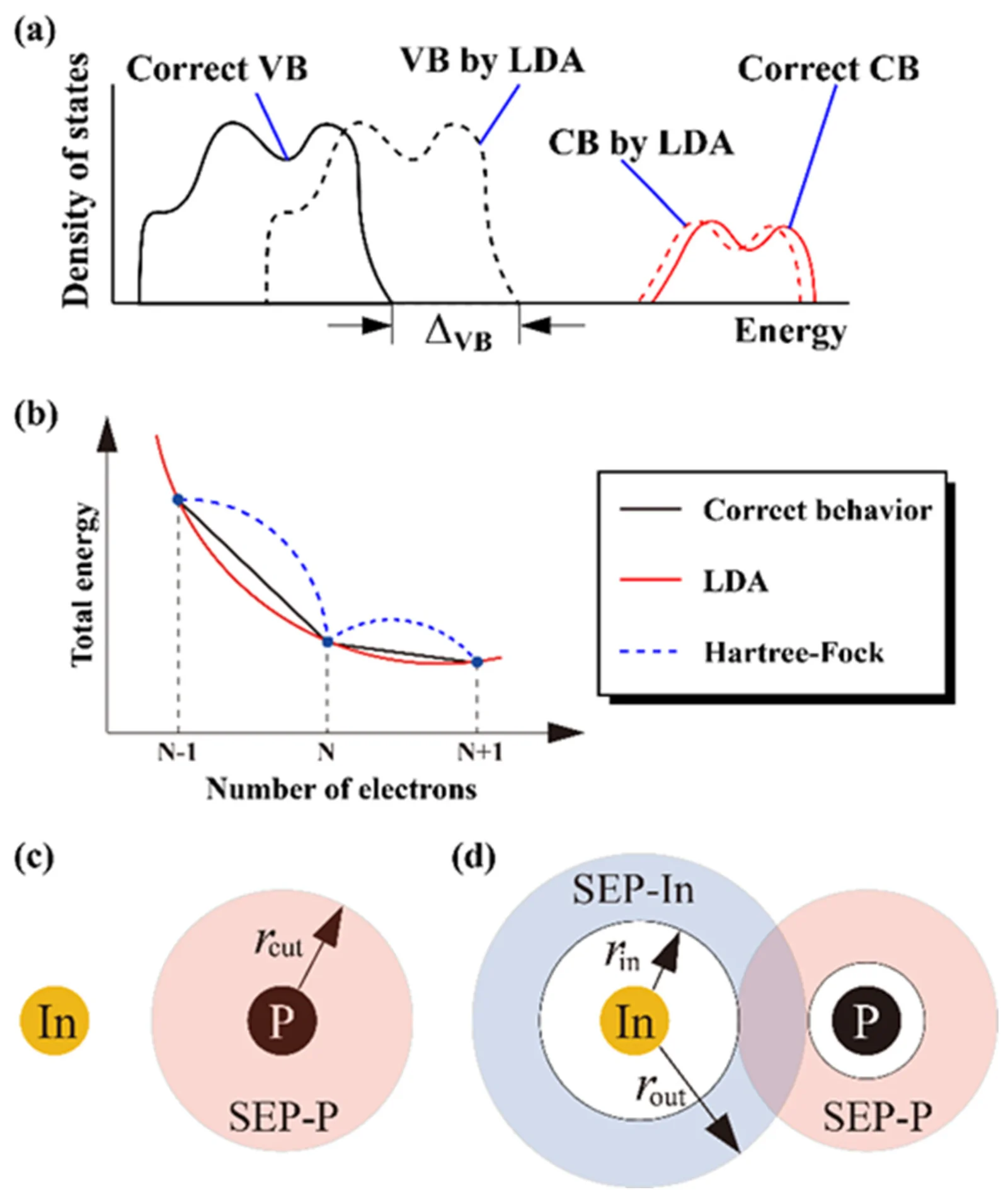
(**a**) Band gap under-estimation in LDA calculations; (**b**) convex and concave behaviors of LDA and Hartree–Fock calculations; (**c**) SEP coverage in InP according to DFT-1/2; (**d**) the SEP coverage in InP from the shell DFT-1/2 perspective.

The intrinsic reason for the “band gap problem” in DFT is related to the lack of clear physical meaning for the Kohn–Sham one-electron eigenvalues ($\epsilon_{i,k}$), which are merely Lagrange multipliers. Provided that the exchange-correlation functional is exact, at zero temperature the highest occupied electronic state should correspond to a negative ionization potential. Except for this special case, no strict physical meaning can be given directly to $\epsilon_{i,k}$ other than the partial derivative of total energy with respect to occupation (Janak theorem [19]). Nevertheless, available LDA and GGA functionals are far from exact, which for instance do not eliminate the spurious electron self-interaction effectively. This renders the “valence band” too high in energy, and the top of the valence band deviates strongly from the negative ionization potential. An over-estimated valence band level inevitably yields a too small band gap, as illustrated in Figure 1a. From a practical point of view, however, one finds that $\epsilon_{i,k}$ resembles the electronic structure in most cases, if one tolerates the “band gap underestimation” as the primary error. Indeed, since DFT under the Kohn–Sham framework has been widely adopted for electronic structure calculations, determining how to resolve the band gap problem has been a long-standing concern.

In the 1980s, it was discovered that the LDA and GGA functionals lack the “derivative discontinuity” (${∆}_{xc}$) [20,21], which causes a systematic underestimation of the semiconductor band gap. Yet, the magnitude of this error is typically much less than the practical band gap underestimation by LDA or GGA. There are two levels regarding the band gap problem by LDA/GGA calculations. On the one hand, their incorrect local or semi-local exchange term yields the self-interaction error (SIE), which greatly raises the valence band level. This contributes to most of the band gap underestimation. On the other hand, the lack of ${∆}_{xc}$ renders a persistent band gap underestimation, even if the exchange-correlation functional is exact under the Kohn–Sham framework.

Yet, in recent years it was claimed that in the generalized Kohn–Sham (gKS) framework [22], the band gap underestimation problem is theoretically solvable [23]. Various hybrid functionals [17,24,25,26] show great application potential in rectifying the band gap problem, in that they incorporate a certain amount of exact (Hartree–Fock) non-local exchange that greatly improves the overall exchange energy of DFT. Most of such errors to be corrected are SIEs [27]. An electron is in parallel spin with itself, and LDA/GGA fails to eliminate the SIE effectively because of their local or semi-local forms for exchange. HSE06 is a range-separated hybrid functional, in which the exact exchange is screened upon the solid-state situation, but it is still non-local and greatly diminishes SIE. This pulls down the valence band to a proper extent. Meanwhile, the conduction band will be slightly raised in an HSE06 calculation compared with a GGA calculation. The non-local exchange is stricter, and to obey the Pauli exclusion principle, the wavefunction of the conduction band state requires greater oscillation, which raises the kinetic energy. This effect is less obvious partly because only 25% of the exact exchange is typically incorporated in HSE06 calculations (denoted by $a_{X}$ = 0.25). To sum up, HSE06 calculations typically predict good electronic structure for a semiconductor, since its valence band is greatly pushed down while its conduction band is slightly raised.

The 25% mixing was initially proposed by Perdew, Ernzerhof and Burke using coupling constant integration [28], where the 1/4 factor emerges through derivative matching arguments. Becke proposed the half-and-half mixing even earlier [24], with $a_{X}=0.5$. If $a_{X}$ is regarded as a tunable parameter, then many solid-state calculations indicate that the optimal $a_{X}$ is usually greater than 0.25 [29]. The improvement in HSE06 over GGA is not only the band gap enhancement, but also globally high accuracy. Take a molecular calculation as an example. LDA and GGA functionals suffer from convexity between integer electron numbers, as shown in Figure 1b, while Hartree–Fock calculations show the opposite concave behavior. Their proper mixing could approach the correct piece-wise linear condition, or the so-called Perdew–Parr–Levy–Balduz (PPLB) condition [20], and potentially recover some derivative discontinuity. Hence, one usually has to adjust the percentage of exact exchange in HSE06 in order to achieve better global performance. This is done by a comparison with experimental data in most cases. In other words, HSE06 calculation can either be carried out in an ab initio way with a 25% exact exchange in the short range, or with the percentage of exact exchange treated as an adjustable parameter.

### 2.2. The Shell DFT-1/2 Method

The DFT-1/2 method, stemming from the Slater half-occupation technique [30] and first proposed in 2008 by Ferreira, Marques and Teles [31], resolves the band gap problem through the self-energy correction perspective. It was claimed that most of the SIE lies with the valence band electrons in a semiconductor; thus, self-energy potentials (SEPs) were introduced in solid-state calculations to pull down the valence band level to its proper location, with respect to the conduction band [31,32]. Compared with the scissor’s operator method that involves an empirical amount of gap correction, DFT-1/2 is nevertheless empirical-parameter-free, since the SEPs were unambiguously derived from atomic calculations, given the definite form of the exchange-correlation functional [33]. Since the SEP is a fairly strong potential, a DFT-1/2 calculation does not yield the correct total energy. Rather, it is a post calculation aiming at rectified electronic structure only. This limits its usage, and charge transition level (CTL) calculation is not possible within DFT-1/2, since it relies on the total energy accuracy.

**Figure 2:**
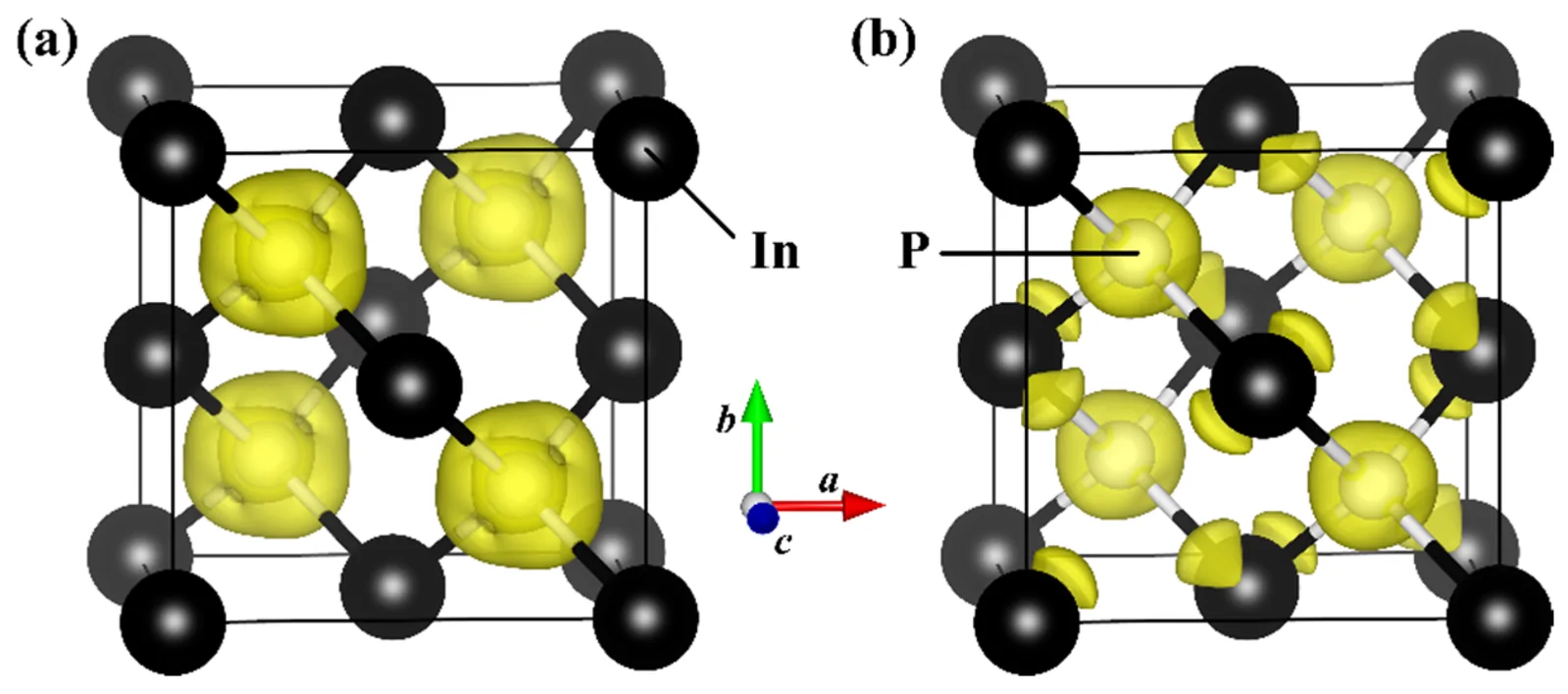
Spatial carrier distribution in InP: (**a**) valence band hole; (**b**) conduction band electron.

In a solid, each SEP should only cover one atom; thus, the SEP is spherically trimmed with a cutoff radius *r*_cut_. An example of InP is demonstrated in Figure 1c. One must attempt various *r*_cut_ values and select the one that gives rise to the maximum band gap. Hence, even *r*_cut_ is non-empirical because it was determined through a variational procedure. Yet, for covalent semiconductors, Xue et al. proposed to use a shell-like trimming function for the SEP [34], which involved both outer cutoff radius (*r*_out_, similar to *r*_cut_ in DFT-1/2) and inner cutoff radius (*r*_in_), both ought to be calculated variationally. This modified method is named shell DFT-1/2. Its benefit is to avoid pulling down the conduction band, which is a quite common artifact in DFT-1/2 when applied to covalent semiconductors. For a typical binary covalent semiconductor like InP, the practical scheme is shell DFT-1/4-1/4, where the two -1/4 e corrections are carried out on In and P, respectively [34], as shown in Figure 1d. The carrier spatial locations, as illustrated in Figure 2, clearly demonstrate that the conduction band electron of InP has a high probability to emerge near both In and P, which is a strong signal of covalent bonding and shell DFT-1/4-1/4 implementation. Hence, in total, four cutoff radii are to be fixed through variational calculation. In this work, the optical radii are $r_{in}=2.5\;Bohr$ and $r_{out}=4.6\;Bohr$ for In, and $r_{in}=1.4\;Bohr$ and $r_{out}=3.2\;Bohr$ for P.

### 2.3. Relation Between Hybrid Functional and Shell DFT-1/2

It is necessary to discuss the similarity and discrepancy between the hybrid functional approach and the shell DFT-1/2 approach. Clearly, both attempt to eliminate the spurious electron self-interaction, but in different fashions. In HSE06, the exchange term is greatly improved to avoid the SIE; thus, the SIE is negligible at the functional level. In shell DFT-1/2, the SIE persists because of the adopted LDA/GGA functional, but the manually involved SEP strives to counteract the electron self-interaction. This is only done for the valence band electrons, but not for other electronic states. Even so, one may envisage fundamentally similar electronic structures obtained through HSE06 or shell DFT-1/2 calculations.

Indeed, in other aspects, shell DFT-1/2 is quite distinct from the hybrid functional approach. For instance, the total energy of shell DFT-1/2 does not make sense. And shell DFT-1/2 itself cannot handle strongly correlated materials, but one has to resort to DFT + U-1/2 [35] or shell DFT + U-1/2 [33,36]. Moreover, shell DFT-1/2 usually also raises the conduction band a bit, but this is merely due to the self-consistent calculation procedure, as explained in a recent publication [37]. However, for III-V semiconductors such as InP, one may attempt the interchange of HSE06 and shell DFT-1/2 for defect calculations. The principle is documented as follows. Since shell DFT-1/2 works very well for band gap correction, it can be used to predict the fundamental gap without any empirical parameter. This process is ab initio, refusing experimental input. The shell DFT-1/2 band gap can be used to tune the percentage of exact exchange in HSE06, upon gap matching. The CTLs may be further calculated using the parameterized HSE06. For density of states or electronic band structure, the more efficient shell DFT-1/2 method may still be used, due to its efficiency and its good matching with the HSE06 results.

### 2.4. Details of Computational Settings

The ground state structure of InP is zinc blende with space group $F\bar{4}3m$, whose primitive cell has yet a rhombohedral appearance ($\alpha =\beta =\gamma =60^{{}^{\circ}}$). In order to minimize the artificial interaction between a defect and its periodic images, we constructed 4 × 4 × 4 supercells with 128 atoms for defect calculation. The types of defects under investigation were In vacancy (V_In_), P vacancy (V_P_), P anti-site defect P_In_, In anti-site defect In_P_, In interstitial In_i_, and P interstitial (P_i_).

Our DFT calculations were carried out within the Vienna Ab initio Simulation Package (VASP) [38,39]. The exchange-correlation energy was approximated by the parameter-free Perdew–Burke–Ernzerhof (PBE) functional [40] of the GGA type. The projector augmented-wave (PAW) method [41,42] was employed to describe the interaction between ionic cores and valence electrons. The valence electrons were 4*d*^10^ 5*s*^2^ 5*p*^1^ for In, and 3*s*^2^ 3*p*^3^ for P. After careful convergence testing, the kinetic energy cutoff for the plane wave basis [43] was fixed to 450 eV. Equal-spacing k-point grids were adopted to sample the Brillouin zones. A 11 × 11 × 11 grid was used for primitive cell calculations, while for the 4 × 4 × 4 supercell with a small Brillouin zone the grid was down-sampled to 4 × 4 × 4. The energy convergence criterion was 10^−5^ eV between two consecutive self-consistent cycles. During structural relaxation, the force criterion was set to 0.01 eV/Å along any direction for each atom. The residual pressure for the primitive cell was kept below 200 MPa along each direction.

The formation energy of a possibly charged (amount of charge being q) defect is calculated using the following formula

$$E_{f}^{q+}=E_{d}^{q+}-E_{0}-\sum_{i}n_{i}\mu_{i}+q\left(E_{v}+E_{F}\right)+E_{\mathrm{corr}}$$

where $E_{d}^{q+}$ is the total energy of the defective supercell (in fact, q can be positive, negative or zero) after relaxation, $E_{0}$ is the total energy of perfect InP in the same supercell size, $\mu_{i}$ is the chemical potential of the *i*th relevant species, and $n_{i}$ is the number of extra atoms of species *i* in the defective supercell. In case the species is subject to removal rather than addition in the defective supercell, we have $n_{i}<0$. $E_{v}$ is the valence band maximum (VBM) position and $E_{F}$ is the system’s Fermi level with respect to VBM. And $E_{\mathrm{corr}}$ is the correction term originating from the limited size of the supercell and especially the image charge effect. The chemical potentials of In and P should satisfy the chemical equilibrium criterion. Under the In-rich condition, the chemical potential of In is set to that in the bulk ground state

$$\mu_{\mathrm{In}}=\mu_{\mathrm{In}}^{\mathrm{bulk}}$$

And the chemical potential of P is derived upon

$$\mu_{P}=\mu_{\mathrm{InP}}^{\mathrm{bulk}}-\mu_{\mathrm{In}}^{\mathrm{bulk}}$$

Under the P-rich condition, the chemical potential of P is set to that in the bulk state

$$\mu_{P}=\mu_{P}^{\mathrm{bulk}}$$

And the chemical potential of In is derived upon

$$\mu_{\mathrm{In}}=\mu_{\mathrm{InP}}^{\mathrm{bulk}}-\mu_{P}^{\mathrm{bulk}}$$

The defective supercell calculations were carried out within the DASP software package (version 2023B) [44]. This package accepts the reference band gap (in our work it comes from the parameter-free shell DFT-1/2 calculation), adjusting the portion of Hartree–Fock exchange in the HSE06 functional [17,18] to maintain the desired electronic structure. The reliable HSE06 total energy was then utilized to calculate the defect formation energy. The artificial electrostatic interaction in periodic cells was corrected using the technique proposed by Freysoldt, Neugebauer and van de Walle (FNV) [45]. The relative dielectric constant of InP was set to 12.5 in the FNV correction scheme.

## 3. Results and Discussion

### 3.1. Electronic Structures of InP

**Figure 3:**
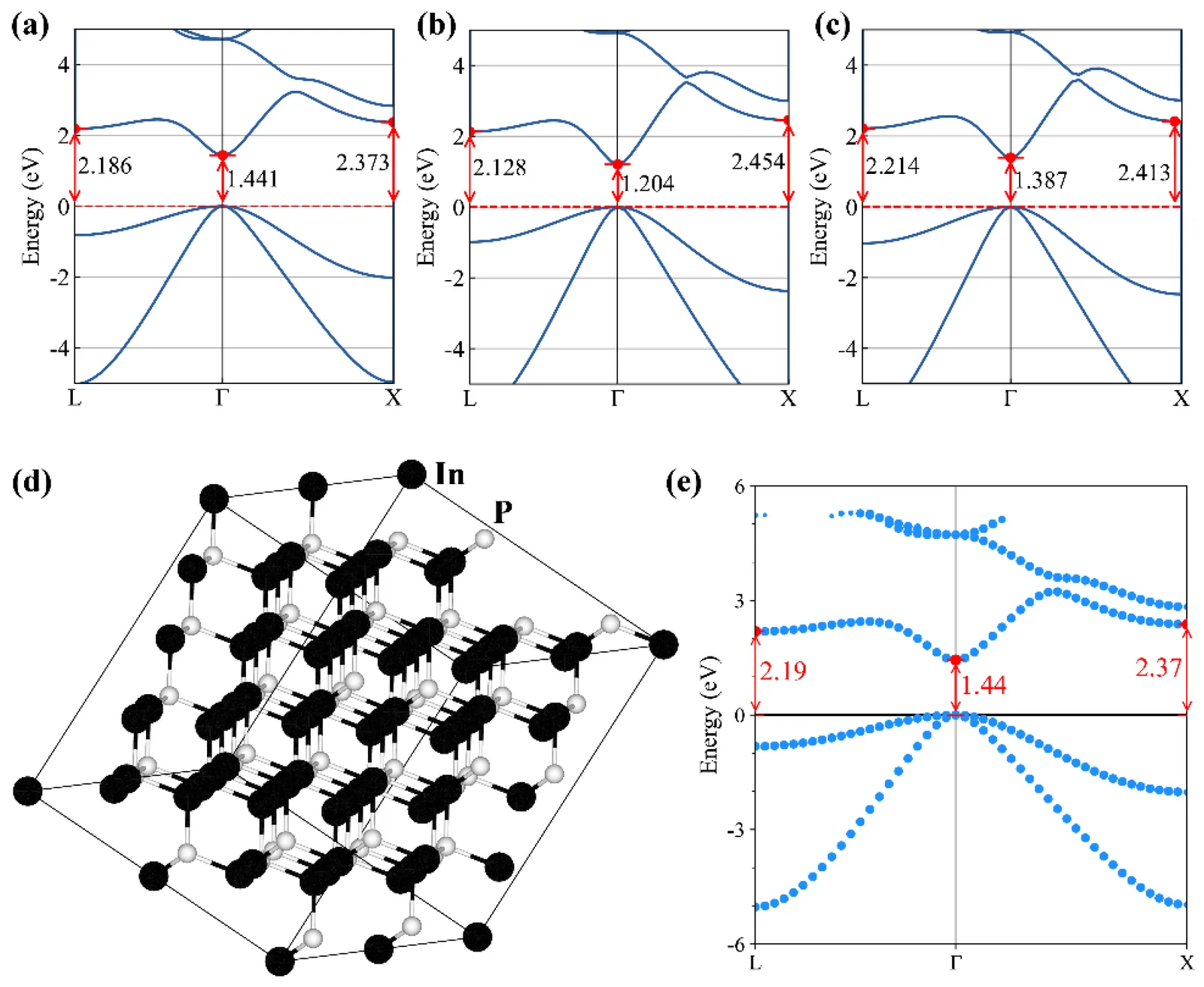
Electronic band structures of InP. (**a**) Shell DFT-1/2 result for the primitive cell; (**b**) HSE06 result with lattice constant optimized by PBE; (**c**) HSE06 result with lattice constant optimized by HSE06; (**d**) atomic structure of an InP supercell; (**e**) shell DFT-1/2 band structure calculated with the supercell.

The shell DFT-1/2 band structure calculated using an InP primitive cell is demonstrated in Figure 3a. The structure was obtained through relaxation by the PBE functional (optimized lattice constant 5.9586 Å). For comparison, the band structure was re-calculated using the HSE06 hybrid functional, on the same structure (Figure 3b). Moreover, the InP lattice parameters were optimized again using HSE06, and the HSE06 band structure under that lattice constant (5.8999 Å) is shown in Figure 3c. The three band structures are quite similar, though shell DFT-1/2 predicts a slightly larger band gap of 1.44 eV. Experimentally, the relation of the InP band gap versus temperature is

(1) $$E_{g}\left(T\right)=1.421-0.00049\frac{T^{2}}{T+327}\;(eV)$$

where T is in units of K. Compared with the zero-temperature extrapolated experimental band gap (1.421 eV), the shell DFT-1/2 result shows very little deviation.

To investigate the band structures of InP supercells, possibly defective, it is necessary to recover the full band structure from very small Brillouin zone calculations. For the 4 × 4 × 4 supercell shown in Figure 3d, the equivalent electronic energy band structure is recovered as the chart in Figure 3e, with an identical band gap value to that in Figure 3a. Moreover, the indirect band gaps at L and X are 2.19 eV and 2.37 eV, respectively. Compared with previous calculations with other methods (Table 1), the band gap accuracy of shell DFT-1/2 is remarkable.

### 3.2. Intrinsic Point Defects

Six common intrinsic point defects in InP are considered in this work, namely the indium vacancy V_In_, phosphorus vacancy V_P_, phosphorus anti-site P_In_, indium anti-site In_P_, indium interstitial In_i_, and phosphorus interstitial P_i_. Shell DFT-1/2 is not feasible for formation energy calculations since its total energy is incorrect, though its band gap is superior to HSE06. To combine the benefits from both shell DFT-1/2 and HSE06, we treated the portion of exact exchange in HSE06 as a parameter, and by fitting to the shell DFT-1/2 band gap, we obtained a ratio of 0.30. Henceforth, the HSE06 calculation with 30% exact exchange for the short-range part will be temporarily named HSE06-0.30.

**Figure 4:**
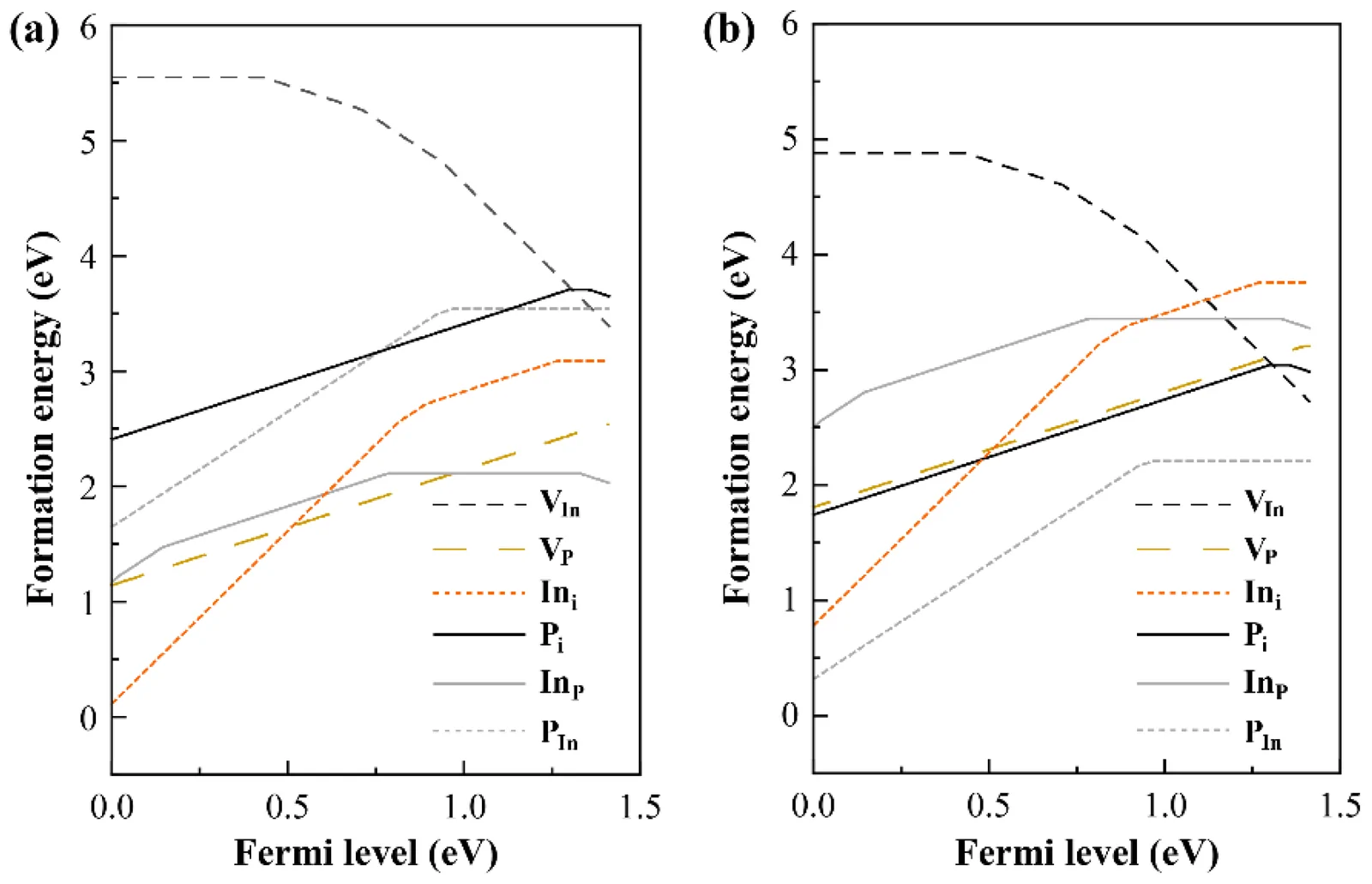
Formation energies of various defects (V_In_: In vacancy; V_P_: P vacancy; In_i_: In interstitial; P_i_: P interstitial; In_P_: In substitution for P; P_In_: P substitution for In), possibly charged, with respect to the Fermi level in InP. (**a**) In-rich environment; (**b**) P-rich environment.

For both In-rich and P-rich growth conditions, the formation-energy curves of these defects as a function of the Fermi level are shown in Figure 4a,b, using HSE06-0.30. All unstable valency states are omitted, and defect states entering the conduction band or valence band are not included. These results provide a direct visualization of the differences in thermodynamic stability among intrinsic defects under various growth environments and electrical doping conditions. Defect formation energy is the key physical parameter for evaluating the tendency of a crystal defect to form spontaneously. A lower formation energy indicates that the corresponding defect is more likely to be generated during crystal growth and annealing, leading to a higher defect concentration and a more pronounced influence on the electrical properties of the material.

From the perspective of chemical environment control, under In-rich conditions the formation energies of V_P_ and In_P_ remain the lowest over the entire Fermi-level range, being significantly lower than those of V_In_ and P_In_. This indicates that an In-rich growth atmosphere strongly promotes the formation of those intrinsic defects with P-missing. Microscopically, this behavior arises because under In-rich conditions, the chemical potential of In is relatively high whereas that of P is relatively low. As a result, the thermodynamic tendency for P deficiency and excess In occupancy in the lattice becomes enhanced, while the energy barriers associated with atomic substitution and vacancy formation are substantially reduced. However, based on existing theoretical and experimental studies, the atomic radius of phosphorus atoms in InP is 20–30% smaller than that of indium atoms. The substitution of a larger indium atom into the lattice position of a smaller phosphorus atom causes significant lattice distortion. Due to atomic size mismatch and severe lattice distortion, the thermodynamic stability of In_P_ is extremely poor, rendering these defects unstable in thermodynamic equilibrium InP crystals under conventional conditions. Existing experimental techniques such as electron paramagnetic resonance (EPR), deep level transient spectroscopy (DLTS) and positron lifetime measurements have also failed to detect characteristic signals of the In_P_ defects [14]. In reality, under indium-rich conditions, only V_P_ serves as the primary intrinsic defect in InP crystals. In contrast, under P-rich chemical conditions, the chemical potential of P atoms increases, whereas that of In atoms decreases. This leads to a substantial reduction in the formation energies of V_In_ and the anti-site defect P_In_, thereby significantly enhancing their thermodynamic stability.

From the viewpoint of Fermi-level regulation, the formation energies of the defects exhibit regular linear variations as the Fermi level shifts, with donor-type and acceptor-type defects showing distinctly opposite responses. Specifically, V_P_ and P_In_ are typical donor defects. As the Fermi level rises, the electron concentration in the system increases, reducing the thermodynamic driving force for defect ionization and electron release. In contrast, V_In_ and In_P_ are acceptor-type defects. An upward shift in the Fermi level facilitates electron capture by these defects, thereby lowering their formation energies and promoting the formation of acceptor-type defects. This trend is fully consistent with the fundamental thermodynamic theory of semiconductor defects.

### 3.3. Defect Charge Transition Levels

**Figure 5:**
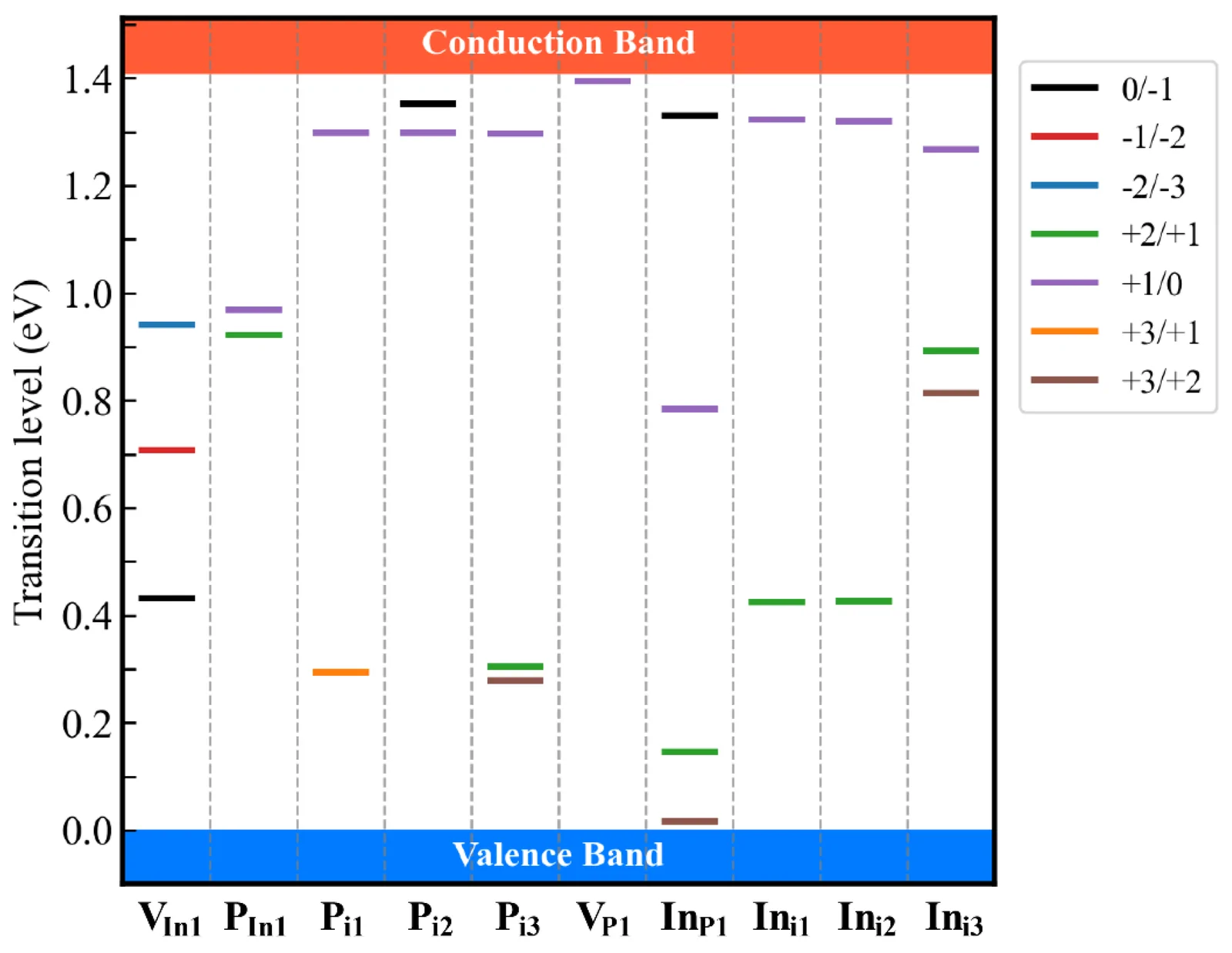
The CTLs of various point defects in InP.

Defect charge-state transition levels (CTLs) are key physical parameters for characterizing the electrical properties of defects, and identifying shallow or deep defect levels. They reveal the thermodynamic transition behavior among various charge states, clarify the mechanisms of carrier capture and release by defects, and provide a microscopic theoretical basis for understanding the intrinsic conductivity, carrier scattering, and leakage behavior of InP-based devices. Figure 5 shows the CTLs within the band gap for the six intrinsic point defects in InP, calculated using HSE06-0.30. The results clearly present the donor/acceptor characteristics, level depths, and charge state transition behaviors of V_In_, V_P_, P_In_, In_P_, In_i_ and P_i_. It follows that the CTLs of various intrinsic defects are distributed quite differently within the forbidden band. Among them, the phosphorus vacancy V_P_ behaves as a typical shallow donor defect. Its (1^+^/0) transition level lies within 0.1 eV of the conduction band minimum. Therefore, electrons can be thermally ionized even at room temperature, continuously supplying free carriers to the conduction band. This microscopic mechanism directly explains the commonly observed unintentional n-type intrinsic conductivity in InP crystals. In particular, under In-rich growth conditions, V_P_ may exist at high concentrations and thus serves as the dominant defect controlling the carrier concentration in InP. In contrast, the indium vacancy V_In_ is a typical deep acceptor defect. Its (1^−^/2^−^) and (2^−^/3^−^) CTLs lie deep in the forbidden band, approximately 0.7–1.0 eV above the valence band maximum. These defective states are highly localized and are difficult to thermally ionize at room temperature. They can, however, readily trap holes and induce stable negatively charged centers, thereby significantly suppressing the p-type conductivity of InP.

Anti-site and interstitial defects exhibit more complex electrical characteristics. The In_P_ defect is amphoteric, possessing both donor and acceptor characteristics. Once the Fermi level is low, it behaves as a donor and can release electrons to compensate the holes. In electron-rich systems with a high Fermi level, it transforms into an acceptor-like defect and captures free electrons, thus exerting bidirectional regulation over the carrier concentration in InP. The CTLs of the P_In_ defect are located near the middle of the forbidden band, indicating that it is a deep-level defect. While it does not participate in room-temperature carrier transport, it can readily act as a carrier recombination center.

In addition to vacancy and anti-site defects, interstitial defects also play an important role in the electrical behavior of InP. Because In_i_ and P_i_ atoms occupy unstable interstitial sites in the lattice, their outer-shell electrons exhibit strong localization, giving rise to multiple deep defect levels within the forbidden band. Metallic In interstitial atoms show donor-like behavior and can additionally release free electrons, further enhancing the n-type conductivity tendency of the system. P interstitial atoms P_i_ also exhibit multi-level donor behaviors and are capability of releasing electrons. Overall, through carrier recombination, interstitial defects influence the dark current and stability of InP-based devices.

### 3.4. Density of States Analyses

While a total energy calculation already involves an electronic structure calculation, recovering the density of states for a defective semiconductor remains difficult for hybrid functional calculations. This is because, while a less dense k-mesh seems sufficient to sample the Brillouin zone of a semiconductor, it cannot yield a detailed density-of-states result. One may use a relatively small supercell with a dense k-mesh for hybrid functional calculations, but the artificial interactions between neighboring defects will then be a large concern. Hence, the shell DFT-1/2 method was implemented to extract the density of states for defective supercells.

**Figure 6:**
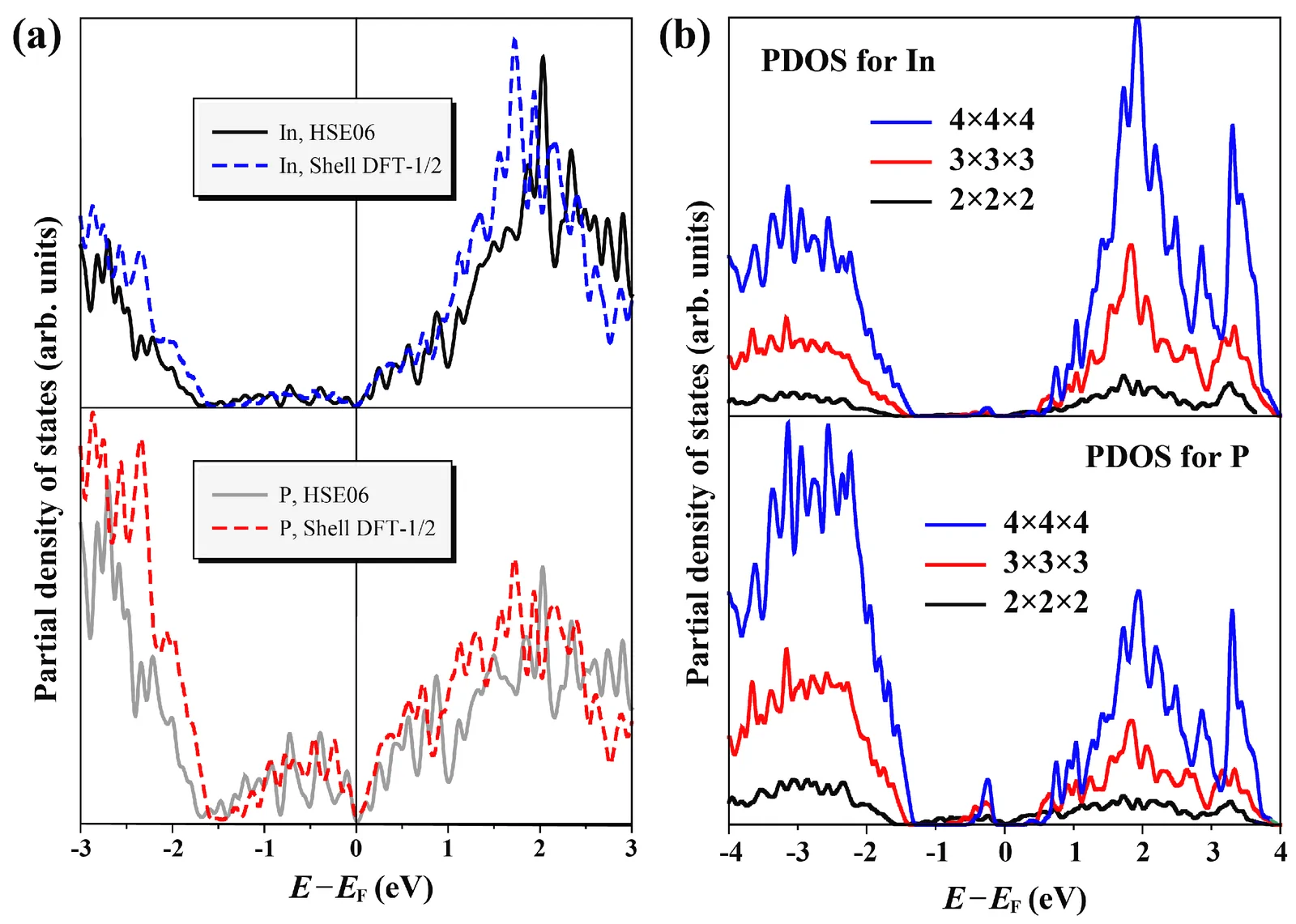
Calculated density-of-states results for InP with P_In_ defect. (**a**) Comparison between HSE06-0.30 and shell DFT-1/2, using 2×2×2 supercell; (**b**) comparison between results from 2×2×2, 3×3×3 and 4×4×4 supercell calculations, using shell DFT-1/2.

**Figure 7:**
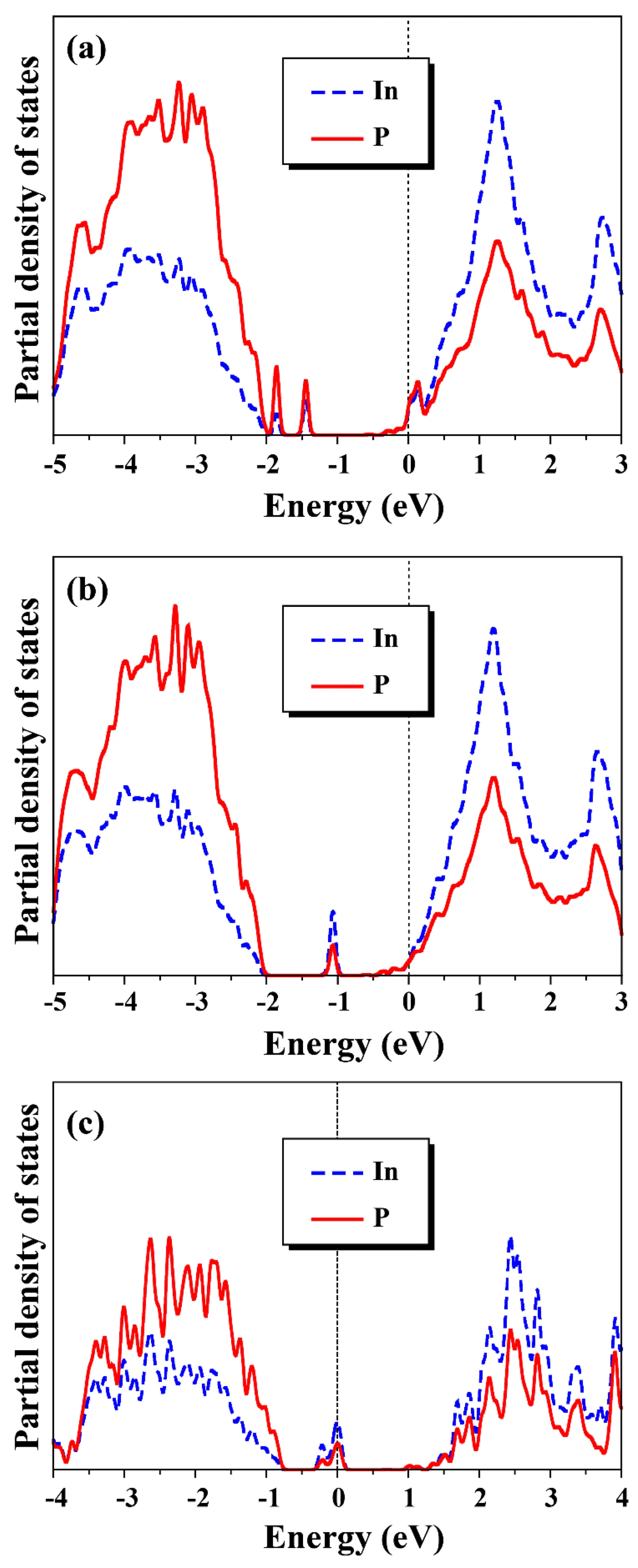
Partial density of states calculated for point defects in InP using shell DFT-1/2. (**a**) P_i_; (**b**) In_i_; (**c**) In_P_.

However, in order to reach a faithful benchmark, it is necessary to compare the shell DFT-1/2 results with those of HSE06. In terms of CTL there is no way to compare the two methods, since shell DFT-1/2 does not predict the correct total energy, but electronic structure provides a suitable platform. Since the percentage of exact exchange was fitted to be 0.30 in our DASP calculation, the same setting was used for the benchmark, and we denote the method as HSE06-0.30. The P_In_ defect was selected as an example. To enable the time-consuming HSE06-0.30 calculation, a small 2×2×2 supercell was established for comparison with the shell DFT-1/2 result. The highest occupied molecular orbital (HOMO) (identified as the Fermi level in VASP) is used to align the energies from both results. In other words, both Fermi levels are set to 0 eV. This scheme has been commonly adopted in existing works [53], and the horizontal axis becomes $E-E_{F}$. As shown in Figure 6a, the density of states predicted by shell DFT-1/2 generally fits the HSE06-0.30 result. Hence, since the parameter-fitted HSE06 calculation was based upon the shell DFT-1/2 electronic structure, shell DFT-1/2 may be used to imitate the HSE06 density of states. On the other hand, Figure 6b demonstrates that the supercell size is critical for the defect analysis. While the 3×3×3 supercell result differs much from the 2×2×2 supercell result, it is close to the 4×4×4 supercell result. This reveals that a sufficiently large supercell is extremely important for defect research. We therefore adopted the 4×4×4 supercell, and Figure 7 illustrates the density-of-states analyses for the P interstitial, In interstitial and In_P_. The P interstitial induces two occupied states slightly above the valence band, and the extra valence electrons from the P interstitial push the Fermi level into the conduction band (Figure 7a). For the In interstitial, the Fermi level is also pushed up due to the extra electrons, but the induced defect state is close to the center of the forbidden band, as illustrated in Figure 7b. If the foreign In atom replaces a P atom, as shown in Figure 7c, the In_P_ defect state is similar to the In_i_ case, but the Fermi level does not enter the conduction band. Rather, it lies within the defect state, because the number of valence electrons for In (3) is less than that of P (5). In contrast, Figure 6b shows that the P_In_ defective state is occupied by electrons, demonstrating the greater number of valence electrons in P compared with In.

**Figure 8:**
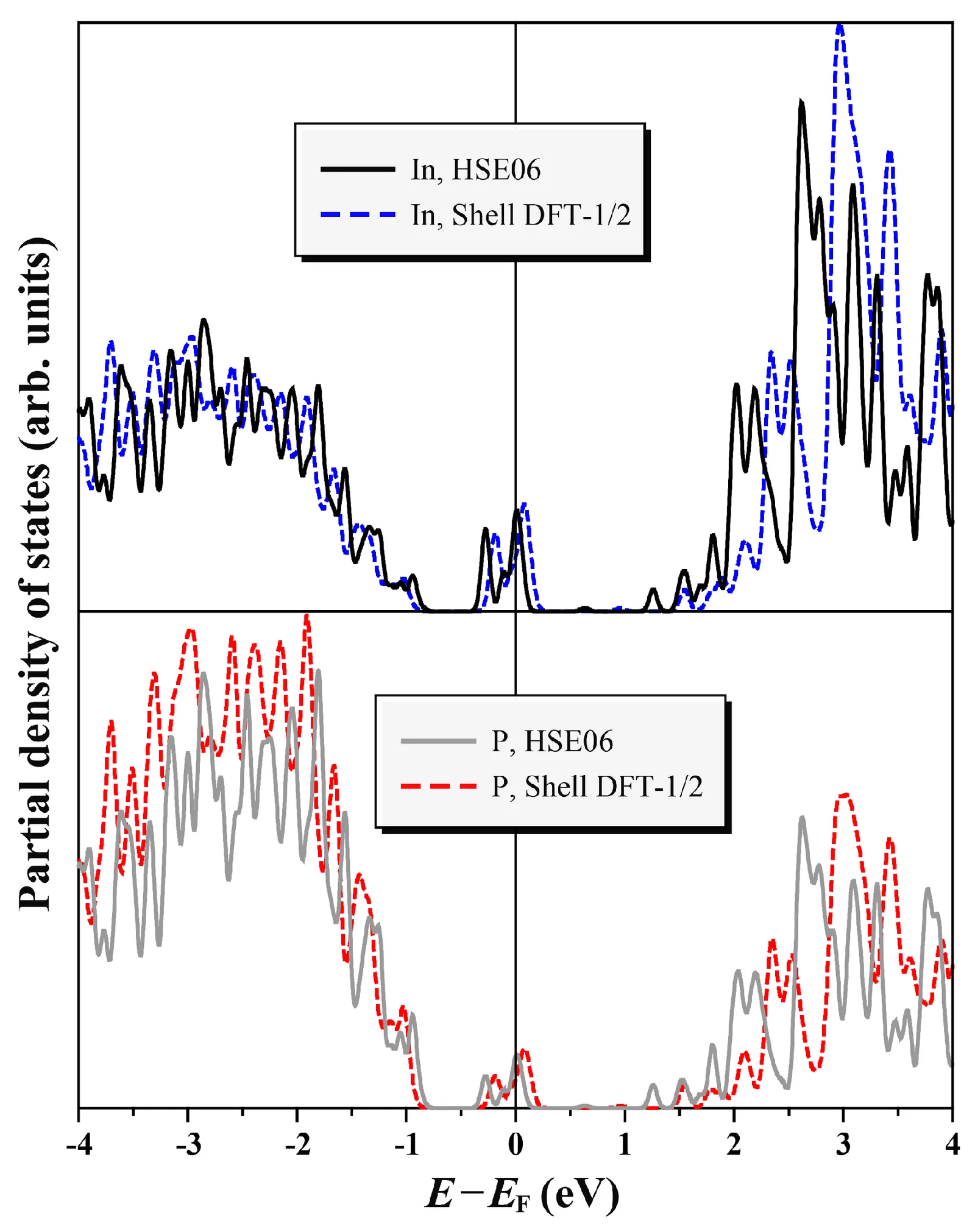
Partial density of states for InP with In_P_ defect using 4×4×4 supercell, with comparison between HSE06-0.30 and shell DFT-1/2 results.

How close is the shell DFT-1/2 density of states with respect to the HSE06 result? It is necessary to go beyond the small 2×2×2 supercell, and to compare the results in more realistic 4×4×4 supercell calculations. The slow computational speed of HSE06 is a hindrance, but it is possible to reduce the density of k points in Brillouin zone sampling. Figure 8 demonstrates a comparison of the partial density of states regarding the In_P_ defect, between HSE06-0.30 and shell DFT-1/2 results. Only a 3×3×3 k point grid was utilized. The overall matching is remarkable, though there are tiny differences regarding the band widths. This is a reasonable distinction, stemming from their intrinsically different treatments of the exchange-correlation term. It nevertheless implies that the shell DFT-1/2 electronic structure is, in general, reliable.

## 4. Conclusions

In conclusion, the shell DFT-1/2 method could generate a satisfactory electronic structure, especially the band gap for widely used covalent semiconductors, but it does not give the correct total energy. To study the defective states and the charge transition levels in semiconductors such as InP, it is feasible to use both shell DFT-1/2 and HSE06 hybrid functional calculations in a complementary way. The shell DFT-1/2 method does not involve empirical parameters; thus, its band gap can be used to tune the portion of exchange in HSE06 (parameter α). The fine-tuned HSE06 calculation gives accurate charge transition levels for the defects. Regarding the electronic structures, including density of states, shell DFT-1/2 and HSE06 yield very similar results. For the sake of efficiency, one could prefer shell DFT-1/2 for electronic structures. The methodology can be extended to defect research in other semiconductors.

## Figures and Tables

**Table 1:** Comparison of zero-temperature InP band gap values from various calculations.

	This Work	Experimental	Other Calculated Values
Band gap (eV)	1.44	1.42 [46]	0.42 ^a^ [47], 0.64 ^b^ [47], 1.58 ^c^ [47], 0.444 ^d^ [48], 1.36 ^e^ [48], 1.55 ^f^ [49], 0.495 ^g^ [49], 0.62 ^h^ [49], 1.32 [16], 1.68 [50]

^a^ LDA calculation from Ref. [47]; ^b^ GGA calculation from Ref. [47]; ^c^ mBJ [51] calculation from Ref. [47]; ^d^ calculated value from Ref. [48]; ^e^ corrected value from Ref. [48]; ^f^ mBJ calculation from Ref. [49]; ^g^ GGA calculation from Ref. [49] using the Wu–Cohen functional [52]; ^h^ GGA calculation from Ref. [49] using the PBE functional.

## Data Availability

Data available on request to the authors.
